# Cell Surface Labeling and Detection of Protein Tyrosine Kinase 7 via Covalent Aptamers

**DOI:** 10.1021/jacs.3c02752

**Published:** 2023-07-20

**Authors:** Savannah Albright, Mary Cacace, Yaniv Tivon, Alexander Deiters

**Affiliations:** Department of Chemistry, University of Pittsburgh, Pittsburgh, Pennsylvania 15260, United States

## Abstract

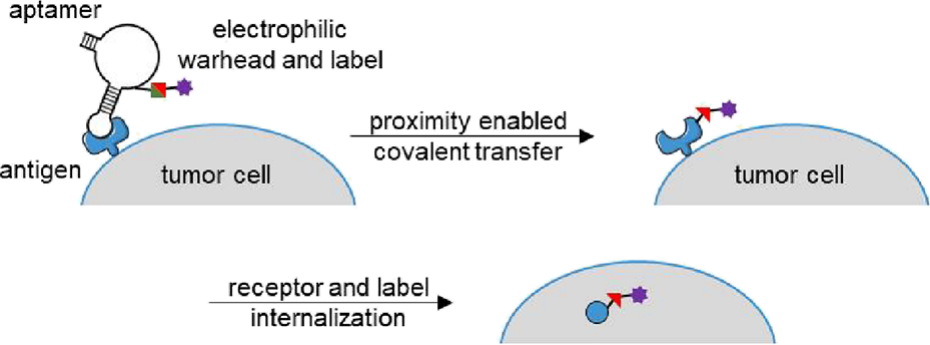

Covalent aptamers are novel biochemical tools for fast and selective transfer of labels to target proteins. Equipped with cleavable electrophiles, these nucleic acid probes enable the installation of functional handles onto native proteins. The high affinity and specificity with which aptamers bind their selected targets allows for quick, covalent labeling that can compete with nuclease-mediated degradation. Here, we introduce the first application of covalent aptamers to modify a specific cell surface protein through proximity-driven label transfer. We targeted protein tyrosine kinase 7 (PTK7), a prominent cancer marker, and demonstrated aptamer-mediated biotin transfer to specific lysine residues on the extracellular domain of the protein. This allowed for tracking of PTK7 expression, localization, and cellular internalization. These studies validate the programmability of covalent aptamers and highlight their applicability in a cellular context, including protein and small molecule delivery.

## Introduction

Aptamers are short, single-stranded oligonucleotides that bind to proteins with affinity and specificity comparable to antibodies.^1^ In vitro and in vivo selection processes have been developed in order to identify aptamers from large libraries of randomized nucleic acid molecules. One such aptamer is sgc8c, a 41-nucleotide DNA molecule that selectively binds specific leukemia cells.^2^ The target of sgc8c is protein tyrosine kinase 7 (PTK7), a catalytically inactive, transmembrane receptor pseudokinase that is implicated in cell survival, growth, and migration.^3,4^ PTK7 has seven extracellular immunoglobulin (Ig) domains that potentially contribute to cell adhesion functions, while its intracellular domain is involved in cell signaling,^5^ specifically via the Wnt and VEGF pathways.^5,6^ High expression levels of PTK7 are a biomarker for numerous cancers, including colon,^7^ non-small-cell lung,^8^ gastric,^9^ and cervical cancer.^10^ Thus, PTK7 is an attractive target for the clinical development of CAR-T cell therapies^11^ and antibody–drug conjugates.^12^

**Figure 1:**
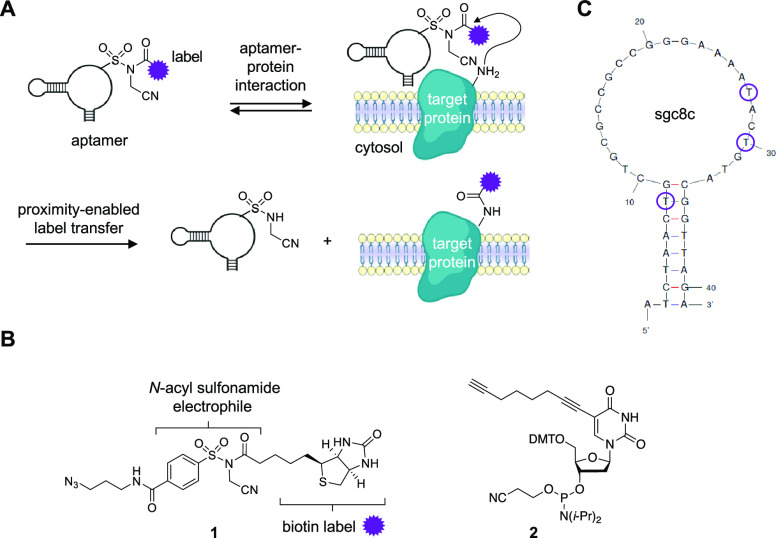
(A) Aptamer-mediated transfer of a chemical motif to the cell surface domain of a protein target. (B) Structures of the alkyne-modified phosphoramidite **2** and the *N*-acyl sulfonamide biotin-transferring electrophilic warhead **1**. (C) Predicted structure of sgc8c, with positions that demonstrated the highest labeling efficiency indicated (8, 27, and 30).

The PTK7-targeting aptamer sgc8c has been employed as a molecular probe for the detection of cancer cells^13−15^ and the delivery of cytotoxic payloads.^16,17^ Although in vivo applications of sgc8c have been reported,^13,18−20^ general limitations of aptamers include their susceptibility to nuclease-mediated degradation,^1^ short half-lives in serum,^21^ and short engagement times with their target protein (high off-rates).^1^ We have recently reported the fast and selective covalent labeling of thrombin through an aptamer modified with cleavable elecrophiles.^22^ Such electrophiles have been pioneered by Hamachi et al. and have found applications in vitro and in vivo using small molecule ligands.^23−26^ Through the use of aptamers, we have expanded the scope of cleavable electrophiles to proteins that cannot be targeted by small molecules. We have found that the most effective approach is to bring an *N*-acyl sulfonamide (NASA) electrophile proximal to nucleophilic residues on the surface of the target protein mediated by an aptamer, allowing for selective label transfer via covalent bond formation (Figure 1A).^22^ The cleaved aptamer subsequently dissociates from the target protein. Covalent aptamers provide the ability to deliver any conceivable functional handle, or even multiple handles, to a target. Furthermore, the covalent bond formation ensures permanent target engagement throughout the lifetime of the protein. Lastly, fast label transfer kinetics establish that protein labeling outcompetes nuclease-mediated aptamer degradation, an important feature for cell-based aptamer experiments.^22^ Overall, these advantages address many of the mentioned shortcomings of traditional aptamers. Here, we report the first application of an aptamer that covalently labels a target protein with high specificity in its native cellular environment.

## Results

After selecting PTK7 as a target, we generated a set of nine electrophilic aptamers by site-specifically incorporating the alkyne-modified phosphoramidite **2** at defined thymidine positions within the sgc8c sequence (5′-A**T**C**T**AAC**T**GC**T**GCGCCGCCGGGAAAA**T**AC**T**G**T**ACGG**TT**AGA-3′) followed by subsequent conjugation with the electrophilic warhead **1** (Figure 1B). The 5-octadiynyl-2′-deoxyuridine phosphoramidite **2** was selected due to its stability during oligonucleotide synthesis and deprotection. Following the reported procedures,^27^ the amidite **2** was synthesized in three steps, which include a Sonogashira coupling of 5-iodo-2′-deoxyuridine and 1,7-octadiyne, a selective 5′-hydroxy dimethoxytrityl (DMT) protection, and conversion to the final phosphoramidite. The aptamers were purified by HPLC prior to their conjugation to **1** via a standard copper-catalyzed [3 + 2] cycloaddition. The biotin-transferring electrophile was synthesized in three steps as well, as previously reported.^22^ Biotin was chosen as a transfer handle because it provides both highly sensitive detection of PTK7 and purification through pull-down with streptavidin resin. The NASA-based electrophile was utilized due its compatibility with cellular environments and its high and selective reactivity toward lysine residues.^25^

**Figure 2:**
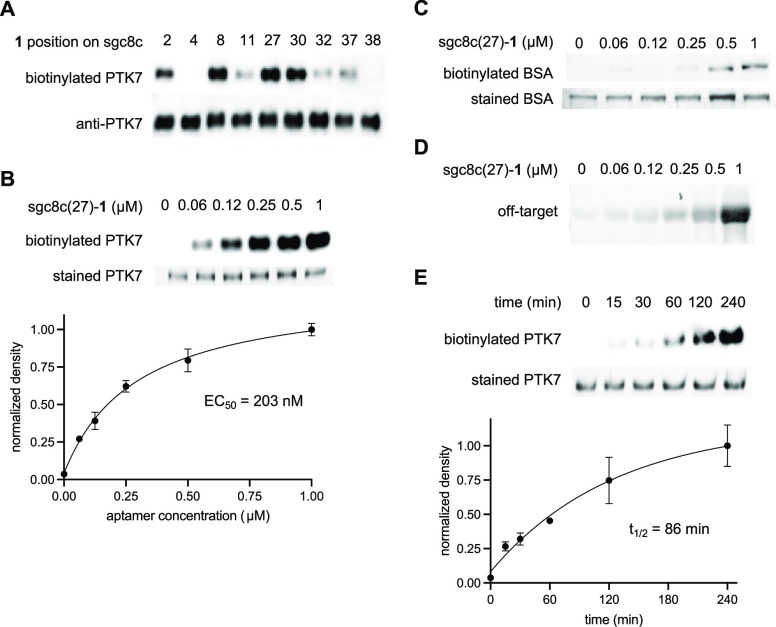
(A) Structure–activity study of aptamers modified with **1** and incubated with PTK7. (B) Dose–response biotinylation of PTK7 by sgc8c(27)-**1**. (C, D) Dose–response analysis of off-target labeling of BSA and serum-supplemented DMEM by sgc8c(27)-**1**. (E) Time course of PTK7 biotinylation. Data points represent averages, and error bars are standard deviations from two to three independent experiments.

The modified sgc8c-**1** aptamers were individually incubated with recombinant PTK7 for 1 h in DPBS supplemented with 5 mM MgCl_2_ and 4.5 g/L glucose (pH 7.4) at 37 °C. The efficiency of biotin transfer was analyzed via simplified immunoblotting using a streptavidin–horseradish peroxidase (SA–HRP) fusion protein (Figure 2A). A distinct structure–activity relationship was observed, with electrophiles installed at positions 8, 27, and 30 (numbered 5′ to 3′) showing the highest PTK7 labeling efficiency. The predicted (UNIFold^28^) stem and loop structure of sgc8c (Figure 1C) includes positions 27 and 30 as part of the loop, whereas position 8 is located on the stem, but is in close proximity to the loop. The results of our SAR study suggest that the loop domain of the aptamer has an increased potential for interactions with PTK7. For subsequent experiments, we moved forward with sgc8c(27)-**1**, though we could have selected positions 8 or 30, and first analyzed the influence of aptamer concentration on PTK7 labeling by conducting a dose–response experiment (Figure 2B, Supporting Figure S1). For this, we incubated recombinant PTK7 (100 nM) with increasing concentrations (up to 1 μM) of the aptamer for 1 h at 37 °C in the aforementioned supplemented DPBS. We observed aptamer-mediated biotinylation of PTK7 at a concentration as low as 62 nM, and it plateaued at 500 nM. Using a non-linear regression fit, we determined an EC_50_ of 203 nM for the label-transferring aptamer. Additionally, we found that aptamer-mediated detection of PTK7 was at least as sensitive as silver staining, a highly sensitive protein stain with a 0.2 ng limit of detection (Supporting Figure S2).

Next, we investigated the kinetics of PTK7 labeling in a test tube, as quick and efficient label transfer could overcome a known limitation of aptamers: their instability to nucleases with an average *t*_1/2_ of about an hour in plasma.^29^ First, the degradation of sgc8c(27)-**1** was analyzed (Supporting Figure S3), which confirmed a *t*_1/2_ of 63 min. A time-course experiment showed that aptamer-mediated PTK7 biotinylation occurred with a labeling *t*_1/2_ of 86 min and plateaued within 4 h (Figure 2E, Supporting Figure S4). These results are promising for translation into a cellular setting since labeling and enzymatic degradation have similar kinetics. It should also be noted that the dissociated aptamer post covalent label transfer is still a substrate for nucleases; however, this degradation has no impact on the covalent protein modification.

With kinetics established, we then examined the selectivity of sgc8c(27)-**1** for PTK7 to ensure successful biotinylation when applied in its native environment. For this, we performed the same labeling reaction described above with bovine serum albumin (BSA), which is a challenging off-target control due its abundance of 60 lysines. We observed aptamer-mediated off-target biotinylation of BSA only at or above a 500 nM aptamer concentration (Figure 2C). To further analyze aptamer specificity, we performed a similar labeling experiment with increasing concentrations of our covalent aptamer in DMEM supplemented with fetal bovine serum (FBS), which also contains nucleases. Again, significant off-target labeling was only observed above 500 nM (Figure 2D), which suggested that 250 nM is the optimal aptamer concentration for PTK7 biotinylation in cells.

**Figure 3:**
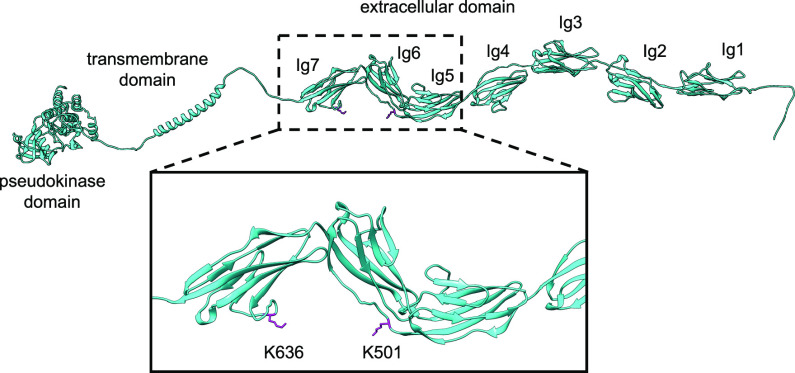
Proposed model of PTK7 (cyan) with biotinylated lysine residues (magenta), as determined by mass spectrometry. The domain structures were predicted by AlphaFold and oriented based on the mass spectrometry results.

Because the sgc8c aptamer was discovered using a cell-based selection approach,^2^ we expected to observe aptamer-mediated biotin transfer to lysines located on the extracellular portion of PTK7 (aa 31–703). After establishing SAR and reactivity on the aptamer side, we interrogated lysine reactivity by mass spec sequencing of the extracellular domain of PTK7 biotinylated with sgc8c(27)-**1** for 1 h at 37 °C (Supporting Figure S5). Results indicated two unambiguous peptides that contained the biotinylated lysines K501 and K636 (Figure 3, Supporting Figure S6).^32^ These lysine residues are located on neighboring, non-homologous Ig domains (Ig6 and Ig7).^30,31^ While the structure of full-length PTK7 has not been reported, only the cytosolic pseudokinase domain,^33^ the biotinylation of these two residues suggests their close proximity in the aptamer-bound state. A resulting hypothesis is that the loop of the sgc8c aptamer binds at or near both of the sixth and seventh Ig domains of PTK7.

**Figure 4:**
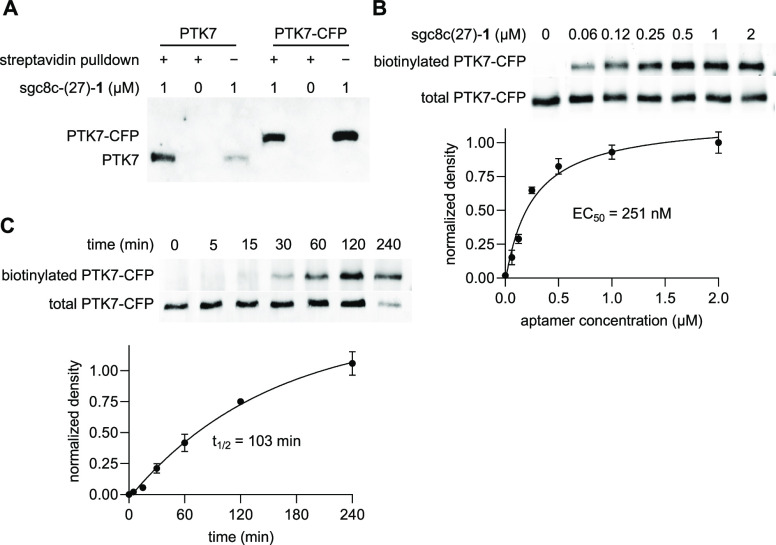
(A) Covalent transfer of biotin from sgc8c(27)-**1** to PTK7 or PTK7-CFP expressed on HEK293T cells and pulled-down with streptavidin resin. (B, C) Dose- and time-dependent biotinylation of HEK293T cells expressing PTK7-CFP, respectively. Data points represent averages, and error bars indicate standard deviation of at least two independent experiments.

To initiate the translation of aptamer-mediated, covalent PTK7 biotinylation from a test tube to mammalian cells, an expression construct for a PTK7-cyan fluorescent protein (CFP) fusion was assembled (Supporting Figure S7). The CFP tag, which enables the visualization of PTK7 localization, was added to the cytosolic^34^ C-terminus of PTK7 to prevent interference with both aptamer binding and label transfer to the extracellular domains. To confirm covalent biotin transfer, cells expressing either PTK7 or PTK7-CFP were incubated for 1 h with sgc8c(27)-**1** (Figure 4A). Pulldowns were performed with streptavidin beads followed by immunoblotting with an anti-PTK7 antibody. Detection of both PTK7 and PTK7-CFP validated successful protein biotinylation of both constructs, proving both covalent modification and that the fusion construct indeed does not disrupt the aptamer–protein interaction. To highlight the importance of the proximity-driven label transfer provided by sgc8c(27)-**1** binding of PTK7, transfected cells were incubated with our previously established covalent thrombin aptamer,^22^ which did not show any cell surface labeling (Supporting Figure S8). These results validated that labeling was not the result of non-specific PTK7–nucleic acid interactions.

After demonstrating the covalent and specific nature of PTK7 labeling, we wanted to determine both the minimum aptamer concentration and the time needed for efficient biotinylation while maintaining PTK7 specificity. In cell-based experiments, labeling was observed within 1 h of incubation at concentrations as low as 62 nM and leveled at approximately 500 nM of sgc8c(27)-**1**, which matched the biotinylation of recombinant PTK7 (Figure 4B, Supporting Figure S9). A non-linear regression was performed using the average band densities to determine an EC_50_ value of 251 nM, similar to that of recombinant protein labeling (203 nM, Figure 2B), and an aptamer concentration of 250 nM continued to be used for subsequent labeling experiments. Even with a 2 μM treatment of sgc8c(27)-**1**, no off-target labeling was observed. The only other bands observed in the immunoblot were also present in the absence of sgc8c(27)-**1** and thus represented proteins that are endogenously biotinylated (Supporting Figure S9). A time-course experiment analyzing biotinylation of cell-surface-expressed PTK7-CFP via sgc8c(27)-**1** (250 nM) showed a *t*_1/2_ of 103 min, which is comparable to test tube experiments (Figure 4C, Supporting Figure S10). Even when the incubation time was extended to 4 h, label transfer was found to be highly specific to PTK7. When the time between label delivery and streptavidin conjugation was extended, cell surface biotinylation of PTK7 was observed up to 16 h after aptamer incubation, though the rhodamine signal was decreased (Supporting Figure S11A). To determine whether this was due to protein degradation or label loss, we performed an SA–HRP blot, increasing the time between label delivery and cell lysis (Supporting Figure S11B). Here, the stable presence of the biotin label was observed up to 16 h followed by a reduced signal at 24 and 30 h. As this exceeds the stability of the aptamer in serum (*t*_1/2_ = 1 h), the biotin signal reflects PTK7 protein turnover. Thus, covalent protein labeling could be used in future studies to analyze protein turnover rates and half-lives. Overall, we concluded that the selectivity of our label-transferring aptamer was maintained in the cellular environment and that cell surface labeling proceeded in a very similar manner (similar EC_50_ and similar *t*_1/2_) to covalent labeling of recombinant protein in a test tube. This was unexpected due to the significantly different biological environments and target engagement being the rate-limiting step, as previously reported for ligand-directed *N*-acyl sulfonamide labeling.^25^ If these observations are generalizable to future aptamer–protein pairs, then they will greatly facilitate optimization and structure–activity relationship studies.

**Figure 5:**
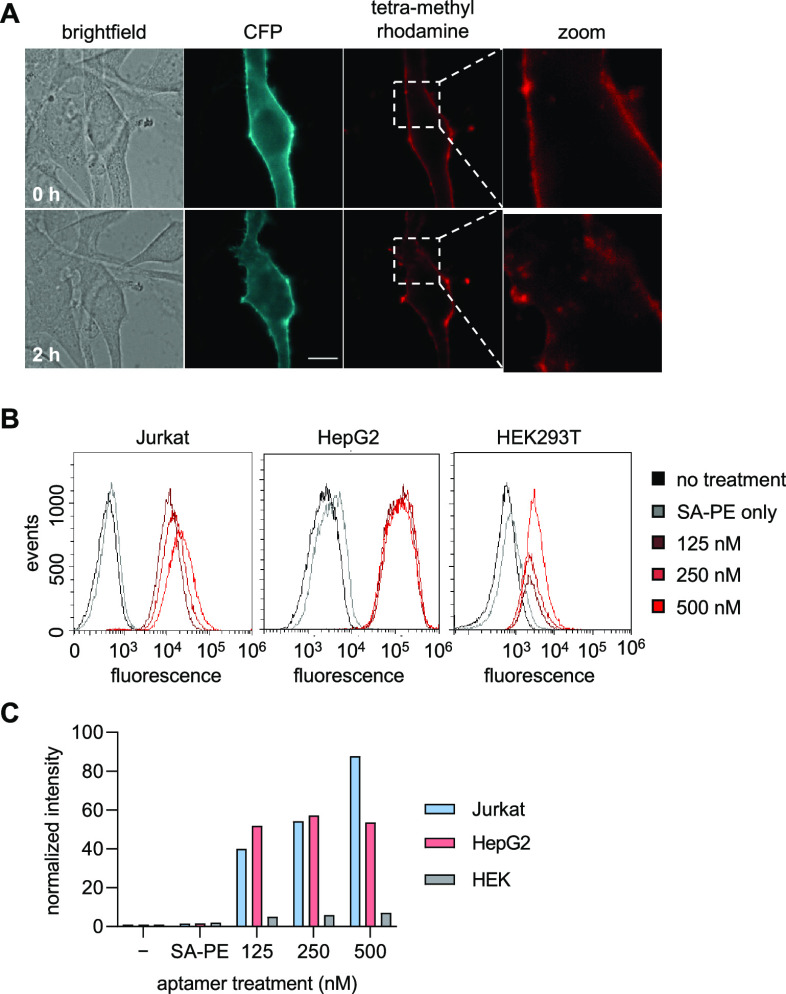
(A) Live cell imaging of NIH3T3 cells expressing PTK7-CFP incubated with sgc8c(27)-**1** and stained with NA-TMR. Cells were incubated at 37 °C for 0 h (top) and 2 h (bottom) after NA-TMR staining. The scale bar represents 10 μm. (B) Flow cytometry of PTK7-positive Jurkat cells (left), PTK7-positive HepG2 cells (middle), and PTK7-negative HEK cells (right) incubated with sgc8c(27)-**1** and conjugated to SA-PE. (C) Quantification of mean intensity of phycoerythrin fluorescence in flow cytometry. Bars represent an MFI of 50,000 recorded events.

We then tested the ability of covalent aptamers to specifically label, detect, and track PTK7 localization via internalization of a cargo. For these studies, NIH3T3 cells, selected for their preferred morphology for imaging, were transfected with PTK7-CFP and incubated with sgc8c(27)-**1** (250 nM) for 1 h. Following aptamer-mediated biotinylation, the cells were briefly incubated for 5 min with neutravidin (NA) labeled with tetramethyl-rhodamine (TMR). Imaging for CFP and rhodamine fluorescence (Figure 5A) showed specific labeling of PTK7-CFP expressing cells, while other cells that are not expressing the CFP fusion are not labeled. Initially, TMR fluorescence was observed exclusively at the cell membrane. However, following a 2 h incubation, we detected internalized TMR fluorescence in endosomes due to the expected recycling of the cell surface receptor (Figure 5A). Live cell imaging revealed that endosome formation started as early as 30 min after labeling (Supplementary Movie 1). The observed localization is consistent with previous studies that show that PTK7 is internalized via caveolin-mediated endocytosis.^36^ These results demonstrate that protein labeling through covalent aptamers can even be applied to the specific cell delivery of cargos, including proteins (e.g., neutravidin) and small molecules (e.g., rhodamine).

Overexpression of PTK7 in acute lymphoblastic leukemia (ALL) is correlated with an increased resistance to apoptosis, making PTK7 a biomarker for cancer detection.^37^ To showcase covalent aptamers as potential tools for cancer diagnostics, we performed labeling experiments in cell lines that endogenously express PTK7. Specifically, we chose Jurkat cells, an ALL lymphocyte cell line, and HepG2 cells, a human hepatoma cell line, as both have been shown to have increased and similar PTK7 levels^38^ that have been correlated to cell proliferation and metastasis.^39,40^ Both cell lines were incubated with increasing concentrations of sgc8c(27)-**1** followed by labeling with a streptavidin–phycoerythrin (SA–PE) construct for 5 min at room temperature. The cells were then analyzed for fluorescence by flow cytometry (Figure 5B,C). Gratifyingly, high levels of phycoerythrin fluorescence were observed only for Jurkat and HepG2 cells, indicating covalent aptamer-mediated detection of endogenous PTK7. Only minimal background labeling was observed in the case of the PTK7-negative HEK293T cells, which again highlights the specificity of this approach. When evaluating the normalized mean fluorescence intensity (MFI) of the labeled cells, a >10-fold increase in MFI was observed for the PTK7-positive cells compared to the PTK7-negative cells. These results suggest the usefulness of our covalent aptamers to detect and modify endogenous PTK7 expressed on the surface of cancer cells.

## Discussion

In summary, we have developed covalent aptamers that can selectively label PTK7 not only in its recombinant form but also expressed in its native, cell surface environment. PTK7 is an important tumor biomarker and a presumed driver in the development and progression of lymphoid, hepatic, and numerous other cancers.^8,37,39,41^ Select reports on the cross-linking of aptamers to cell-surface proteins using electrophilic and photoreactive groups exists;^14,15,42,43^ however, this is the first example of aptamer-mediated selective and efficient transfer of a small molecule label. The labeling reaction is fast, as fast as nuclease-mediated aptamer degradation, and occurs at low aptamer concentrations. Biotin transfer was observed to select lysine residues, and cell-surface-modified PTK7 was labeled with streptavidin fluorophores. This enabled the detection of PTK7 localization and translocation into the cell. Furthermore, we demonstrated specific detection of PTK7-positive acute lymphoblastic leukemia and hepatocarcinoma cancer cells by flow cytometry. Taken together, these results lay the foundation for the application of covalent aptamers as potential cancer diagnostics and therapeutic agents. For example, they can be used in the delivery of multiple fluorophores to cancer biomarkers or the capture of biomarker-expressing cells resulting in more robust diagnoses. Additionally, through the targeted delivery of drug cargos to cancer cells, enhanced therapeutic efficacy could be achieved with covalent aptamers. Here, we have already demonstrated the delivery of a protein and a fluorophore specifically to PTK7-positive lymphoma and hepatoma cells.

## Supporting Information Available

Live cell imaging revealing endosome formation starting as early as 30 min after labeling (MP4)
Experimental details, methods, materials, and supporting figures showing Western blot analysis dose-dependent labeling of PTK7 by sgc8c(27)-**1**, titration of PTK7 for detection by sgc8c(27)-**1**, degradation of ^32^P-labeled sgc8c(27)-**1** in human plasma over time through native-PAGE separation and radiography and quantification of the percentage aptamer remaining as determined by band intensity, Western blot analysis of a labeling timecourse in triplicate, in-gel analysis of PTK7 and biotinylated PTK7 for MS analysis, peptide spectra from MS/MS experiments, plasmid map of pcDNA3-PTK7-CFP for mammalian cell expression, SA-HRP and anti-GFP Western blots of lysate from HEK293T cells transfected with pcDNA3-PTK7-CFP and treated with the biotin-transferring thrombin aptamer TBA(3)-**1** (250 nM) for 1 h, full SA-HRP and anti-GFP Western blot and SA-HRP and anti-GFP Western blot of lysate from HEK293T cells transfected with pcDNA3-PTK7-CFP and treated with increasing amounts of of sgc8c(27)-**1** for 1 h, full SA-HRP and anti-GFP Western blots and SA-HRP and anti-GFP Western blots of lysate from HEK293T cells transfected with pcDNA3-PTK7-CFP and treated with 250 nM of sgc8c(27)-**1** for increasing amounts of time, images of NIH3T3 cells expressing PTK7-CFP incubated with sgc8c(27)-**1** for 1 h followed by staining with NA-TMR either instantaneously (0 h) or after 16 h and SA-HRP and anti-GFP Western blot analysis of the persistence of biotinylated PTK7 over time, and list of primers used to generate DNA constructs (PDF)
