# Supplementary material for: Cell Surface Labeling and Detection of Protein Tyrosine Kinase 7 via Covalent Aptamers
Source: J Am Chem Soc. 2023 Jul 20;145(30):16458–63. doi: 10.1021/jacs.3c02752 (PMC10401710; doi:10.1021/jacs.3c02752)
Supplement: Supplementary file 2 — ja3c02752_si_002.pdf [file ja3c02752_si_002.pdf]

Supporting Information

Cell Surface Labeling and Detection of Protein Tyrosine Kinase 7 via Covalent Aptamers

Savannah Albright, Mary Cacace, Yaniv Tivon, and Alexander Deiters*

Department of Chemistry, University of Pittsburgh, Pennsylvania 15260, United States

Supporting Methods

Oligonucleotide Synthesis. Oligonucleotide syntheses were performed using standard β-cyanoethyl phosphoramidite chemistry on an Expedite 8909 DNA/RNA Synthesizer (Distribio, NY, USA). Aptamers were synthesized on a 200 nanomole scale using 500 Å derivatized CPG solid phase supports obtained from Glen Research (VA, USA) using a 5'-dimethoxytrityl (DMTr)-OFF approach. Synthesis cycles with coupling times of 2 and 10 minutes were used for both the unmodified and the modified phosphoramidite (**2**) respectively, at 0.07 M concentration. Coupling efficiency was monitored by DMTr cation release after each deprotection step – no significant loss was observed following modified phosphoramidite addition to the oligonucleotide.

Reagent and phosphoramidite list (Glen Research, VA, USA). dA-CE phosphoramidite (10-1000), Ac-dC-CE phosphoramidite (10-1015), dmf-dG-CE phosphoramidite (10-1029), dT-CE phosphoramidite (10-1030), tetrahydrofuran/2,6-lutidine/acetic anhydride (40-4010), 5-ethylthio-1H-tetrazole (ETT, 30-3040), 0.02 M iodine in tetrahydrofuran/pyridine/water (88:10:2)(40-4032), 3% dichloroacetic acid in dichloromethane (40-4040), and anhydrous acetonitrile (40-4050).

Oligonucleotide deprotection. Cleavage of the oligonucleotide from the solid support and subsequent deprotection were carried out with 1 mL of 2 N ammonia in methanol at room temperature for 1 h. Following, concentration *in vacuo,* the clear oil was resuspended in 1:1 methylamine in ammonium hydroxide (1 mL) and heated at 65 °C for 15 min. The solution was then concentrated *in vacuo* prior to ethanol precipitation.

Ethanol precipitation of modified oligos. Oligonucleotide precipitation was achieved through the addition of sodium acetate (3 M, pH 5.2, 0.1-times the volume of the oligonucleotide solution) and 100% ethanol (3-times the volume) to the oligonucleotide solution (10-200 μM, 1V) in milliQ water. The solution was vortexed and cooled at –80 °C for 30 min and subsequently pelleted (13.2 rpm, 15 min, 4 °C). The supernatant was discarded, and the pellet was washed with 70% ethanol (3-times the volume) and pelleted as described above. The supernatant was removed, and the pellet was resuspended in milliQ water. The oligonucleotide concentrations were determined by a NanoDrop Spectrophotometer (ND1000). Oligonucleotide purity was analyzed by HPLC, using a gradient of 5-30% buffer B over 30 min at a flow rate of 1 mL/min on an ACE oligo C18 column (4.6 x 100 mm, 3 μM particles). The buffers used were 0.1 M TEAA (Buffer A) and ACN (Buffer B). The column oven was set to 40 °C .

Conjugation of the azide-tethered electrophile to the alkyne-modified oligonucleotide. Synthesized oligonucleotides containing the alkyne modification were subjected to a [3+2] cycloaddition (click) protocol. Reactions were carried out in 1.7 mL centrifuge tubes. To 50 μL of the alkyne-bearing oligo (20-200 μM in milliQ water) was added 5 μL of triethylammonium acetate

buffer (2 M, pH 7, Glen Research) and DMSO (55 µL) before the solution was vortexed. Following 5 µL of the azide electrophile (10 mM in DMSO) was added and the reaction mixture was vortexed. To the resulting solution was added 12 µL of a freshly made ascorbic acid solution (5 mM in deionized water) and the reaction mixture was vortexed again. Argon gas was bubbled through the reaction mixture via a needle for thirty seconds and the tube cap was quickly closed. Lastly, 6.5 µL of copper (II)-tris[(1-bennzyl-1*H*-1,2,3-triazol-4-yl)methyl]amine (TBTA, 10 mM in 55% DMSO) was quickly added, the reaction mixture was capped, vortexed, and incubated at room temperature for 1 h. Reaction progression was monitored by HPLC using the method/conditions described previously. Upon reaction completion, the click reaction was isolated following an ethanol precipitation. If the click reaction did not proceed to completion after 4 h, the oligonucleotide (following ethanol precipitation) was purified by HPLC and dried on a speedvac (ThermoFisher). The dried oligonucleotides were then resuspended in 50 µL of milliQ water and their concentrations were determined by NanoDrop (ND1000).

Laemmli loading buffer recipe (6X). Tris buffer (1 M, pH 6.8, 15 mL), SDS (6 g), glycerol (30 mL), 2-mercaptoethanol (15 mL), and bromophenol blue (0.1 g) were combined in a 50 mL conical tube. Deionized water was added to 40 mL and the solution was vortexed until the solids dissolved before the remaining 10 mL was added. The buffer was aliquoted and stored at –20 °C .

Aptamer-mediated biotin transfer to recombinant protein. Recombinant PTK7 (R&D Biosystems, NC2081082) and BSA (ThermoFisher, AAJ1085722) were diluted to 10 µM and 100 µM, respectfully, in DPBS supplemented with 5 mM $MgCl_2$ and 4.5 g/L glucose, pH 7.4 (wash buffer). The PTK7 solution (2 µL) was further diluted into DPBS (16 µL) or DMEM supplemented with 10% FBS (16 µL). If performing a selectivity experiment, PTK7 was diluted with DPBS (14 µL) and supplemented with a BSA solution (2 µL) for final concentrations of 100 nM and 1 µM, respectively. A 10 µM aptamer solution was made in MilliQ water. For additional aptamer concentrations, the 10 µM stock was further diluted to achieve 5, 2.5, 1.25, 0.63 µM (10X) working stock solutions. Labeling was initiated by the addition of the aptamer solution (2 µL) to the PTK7 solution to a final reaction volume of 20 µL. Reactions were incubated at 37 °C for the indicated amount of time. Reactions performed in serum were supplemented with 0.1 mg/mL yeast tRNA (Fisher).

Analysis of biotinylated proteins *in vitro.* Following aptamer-mediated biotin transfer, reaction samples (20 µL) were heated in Laemmli sample buffer (4 µL) for five minutes at 95 °C on a heat block. Samples (10 µL) were loaded onto two 6% SDS-PAGE gels, and separated via electrophoresis (60 V 20 min, 140 V 1.25 h). Proteins were transferred (80 V, 1.75 h) to a PVDF membrane (GE Healthcare) and the membrane was incubated in blocking buffer (5% BSA in TBS with 0.1% [v/v] Tween 20 (TBST)) for 1 h at room temperature. The blots were probed with streptavidin-HRP (SA-HRP, 1:10000 dilution, Thermo Fisher, N100) in 1X TBST (10 mL) at room temperature for 1 h while rocking. Blots were then washed three times with 1X TBST (10 mL, 10 min) at room temperature while rocking. Chemiluminesence was developed using SuperSignal West Pico Chemiluminescent Substrate (ThermoFisher, 8 mL) and imaged on a ChemiDoc Imaging System (BioRad) using automated exposure settings. To visualize the total protein loaded, gels were developed with silver stain (Pierce, ThermoFisher, 24162) according to the manufacturer's protocol and imaged on ChemiDoc Imaging system using the automated exposure settings. For quantification (**Figure 2B,E**) bands were integrated using ImageJ by drawing rectangles around lanes and analyzed using the plot lanes function, which includes a background subtraction. Band integrations were normalized to the total protein in the loading control.

MS/MS analysis of biotinylated PTK7. Recombinant PTK7 (2.4 µL, 4.2 µM) was added to DPBS (16 µL, pH 7.4) supplemented with 5 mM $MgCl_2$ and 4.5 g/L glucose. A 10 µM aptamer solution was made in MilliQ water. A non-treated and treated sample were prepared: for the treated sample, labeling was initiated by the addition of the aptamer solution (2 µL) to the PTK7 solution (18 µL) to achieve a final volume of 20 µL containing 500 nM PTK7 and 1 µM of aptamer. For the non-treated sample, MilliQ water (2 µL) was added to the PTK7 solution (18 µL). Reactions were incubated at 37 °C for 1 h. At completion, reactions were heated in Laemmli sample buffer (4 µL) for five minutes at 95 °C on a heat block. Samples (15 µL) were loaded onto a 6% SDS-PAGE and separated via electrophoresis (60 V, 20 min, 140 V, 1.25 h). Proteins were visualized with a silver stain (mass spec compatible, ThermoFisher, 24612) following the manufacturer's instructions. The PTK7 bands were excised using a scalpel, placed into LoBind Microcentrifuge Tubes: Protein (Fisher, 13-698-794), and covered with HPLC grade water (1 mL, Fisher, 60-026-52). The gel slices were shipped to the Wayne State Proteomic Facility for protein sequencing.

Plasmid construction. Top10 chemically competent cells were used for all cloning. See **Supporting Table 1** for a list of primers used. Maps of cloned plasmids are presented in **Supporting Figure 8**. The CFP coding sequence was PCR amplified from pLyn-FKBP-FKBP-CFP (Addgene, 20149) using primers P1 and P2 and cloned into the pcDNA3-PTK7-VSV (Addgene, 65250), which was amplified using primers P3 and P4. All PCRs were performed using Phusion (ThermoFisher, F534L) according to the manufacturer's protocol. PCR products were gel purified (ThermoFisher, K0692) according to the manufacturer's protocol. The two-fragment Gibson assembly reaction was set up using 100 ng of the pcDNA3-PTK7-VSV amplicon and a 2:1 ratio of the CFP amplicon following a previously reported protocol.[1] The reaction was incubated at 50 °C for 1 h and then treated with Dpn1 (NEB) overnight prior. To transform cells, a 50 µl aliquot of Top10 cells was thawed on ice in a 1.7 mL microcentrifuge tube and combined with 5 µL of the Gibson assembly mixture. The cells were mixed by gently tapping the side of the tube and the mixture was incubated on ice for 30 minutes. The cells were heat shocked by incubating the tube in a hot water bath (42 °C, 30 sec), following by addition of 200 µL of SOC media (VWR, 100219-988). The tube was incubated at 37 °C with shaking at 250 rpm for 40 min. The entire content of the transformation mixture was plated on 10 mL of LB agar supplemented with 10 µL of a 100 mg/mL ampicillin stock and incubated overnight at 37 °C. One colony was inoculated into 5 mL of LB broth supplemented with 5 µL of a 100 mg/mL ampicillin stock and grown overnight with shaking (37 °C, 250 rpm), followed by DNA miniprep (Thermo Scientific, K0503). The construct was confirmed by Sanger Sequencing (Azenta) using the available "EGFP-N" forward (P5) and "BGHR" reverse (P6) sequencing primers.

Cell culture maintenance. All cell culture experiments were performed in a sterile laminar flow hood. HEK293T and NIH3T3 cells were maintained in Dulbecco's Modified Eagle Medium (DMEM) supplemented with 10% (v/v) fetal bovine serum (FBS) and 1% (v/v) penicillin/streptomycin (p/s) at 37 °C with 5% CO_2. Jurkat cells were maintained in RPMI-1640 medium supplemented with 10% (v/v) FBS and 1% (v/v) p/s. HepG2 cells were maintained in Eagle's Modified Eagle Medium (EMEM) supplemented with 10% (v/v) FBS and 1% (v/v) p/s. Cells were used between passage number 6 and 30. Cell lines were tested for mycoplasma contamination using the MycoScope PCR Detection Kit (Genlantis, MY01100) every 4 months.

Protein labeling and western blot analysis. Cells (50,000 per well in 250 µL of antibiotic-free DMEM) were seeded into a 48-well plate. When cells reached 80-90% confluency, transfection reagents were prepared by combining 400 ng of plasmid with 0.8 µL of P3000, 0.8 µL of Lipofectamine 3000 (ThermoFisher), and Opti-MEM transfection media (Thermo Scientific) to a total volume of 20 µL per well. This was scaled up according to the desired number of transfected wells to minimize pipetting steps. Cells were incubated at 37 °C with 5% CO_2 overnight. After

imaging CFP fluorescence using a Zeiss Axio Observer Z1 with an Andor Zyla 4.2 camera, Plan-Apochromat 63x/1.4 objective, and the CFP filter (47 HE, ex. BP 436/20, em. BP 480/40), cells were washed three times with 200 µL of room temperature DPBS containing 5 mM $MgCl_2$ and 4.5 g/L glucose (wash buffer). Labeling was performed by incubating cells with 100 µL of solution of the indicated concentration of sgc8c(27)-**1** in DPBS containing 5 mM $MgCl_2$, 4.5 g/L glucose, and 0.1 mg/mL yeast tRNA (binding buffer), for the indicated time at 37 °C. For label stability studying, cells were washed once with 100 µL of wash buffer and incubated in 250 µL of DMEM with 10% FBS for the indicated time. Cells were then washed three more times with 100 µL of wash buffer, lysed with 75 µL of RIPA buffer (Fisher) containing 100x HALT protease inhibitor (ThermoFisher) by shaking on ice for 20 minutes. Lysates were centrifuged at 4,000 x g for 10 minutes at 4 °C to pellet any cell debris. The supernatant was transferred to a fresh 1.7 mL microcentrifuge tube and stored at –80 °C until further use.

Streptavidin pulldown. A 50% streptavidin sepharose bead slurry (10 µL, GE Healthcare, 45-000-279) was pipetted into a 0.65 mL tube. The resin was washed three times with 100 µL of PBS, followed by centrifugation at 200 x g for 30 seconds. The supernatant was removed, and crude lysate (60 µL) was added, followed by brief vortexing. The suspension was incubated for 1 hour at 4 °C with shaking. Following incubation, non-biotinylated proteins were removed with 3 washes with 100 µL of PBS (0.1% SDS, 1% NP-40), 2 washes with 100 µL of PBS (0.1% SDS, 1% NP-40, 0.4 M NaCl), and 1 wash with 100 µL of PBS. After each wash, the suspension was centrifuged at 200 x g for 30 seconds and the supernatant was removed. Biotinylated protein was eluted by heating for 15 minutes at 95 °C in 10 µL of 6X Laemmli buffer containing 8% (v/v) 2-mercapto ethanol and 10 µL of a 6 mM biotin solution in water.

SDS-PAGE and western blot analysis of biotinylated proteins. Crude lysates (25 µL) were heated in Laemmli buffer (5 µL) for 10 minutes at 95 °C. SDS-PAGE 2-step gels (6% top/10% bottom (v/v)) were loaded with 12 µL of each sample, which were separated via electrophoresis (60 V, 20 min then 150 V, 1.25 h). Afterwards, proteins were transferred (80 V, 1.5 h) to a PVDF membrane (GE Healthcare) and the membrane was blocked in blocking buffer (5% in 1X TBST]) for 1 h. Blots were probed for biotinylation with streptavidin-HRP (Thermo Fisher, 1:10000 dilution in TBST) at room temperature for 1 h with rocking. Anti-GFP and anti-GAPDH control blots were incubated with anti-GFP rabbit polyclonal (Proteintech, 50430-2-AP, 1:5000 dilution in TBST) and anti-GAPDH rabbit polyclonal (Proteintech, 10494-1-AP, 1:5000 dilution in TBST) antibodies, respectively, at 4 °C overnight with rocking. Blots were washed with 5 mL of TBST for 5 minutes at room temperature with rocking before incubating with secondary goat anti-rabbit antibody (Proteintech, SA00001-2, 1:5000 dilution in TBST) for at room temperature for 1 h with rocking. Chemiluminescence was developed using SuperSignal West Pico Chemiluminescent Substrate (Thermo Scientific) and imaged on a ChemiDoc MP Imaging System using automated exposure settings. For quantification, bands were integrated using ImageJ by drawing rectangles around lanes and analyzing using the plot lanes function. Band integrations were normalized to the loading control.

Aptamer stability study through radiography. For ^{32}P labeling, aptamer (4 µL, 10 µM) was mixed with 10x T4 PNK buffer (NEB, 2 µL), gamma ^{32}P-ATP (PerkinElmer, 3000 Ci/mmol, 1 µL), T4 PNK (NEB, 1 µL), and nuclease free-water (12 µL), and incubated at 37 °C for 1 h. The isotope labelled ssDNAs were purified using Microspin™ G-25 columns (GE Healthcare) and used for the stability study. Samples (3 µL) were incubated in human plasma (9 µL, Millipore Sigma P9523) for indicated periods of time. The samples were then diluted with 5x PAGE loading buffer (2.4 µL) and separated by 16% (v/v) PAGE gel electrophoresis (120 V, 1 h). The gel was exposed to a phosphor screen overnight and scanned with a Typhoon FLA7000 IP Phosphorimager (GE Healthcare). Bands were integrated using ImageJ by drawing rectangles around the lanes and

analyzing them using the plot lanes function. Band integrations were normalized to the 0 min time point.

Live cell fluorescence imaging. Cells (30,000 per well) were seeded into a 8-well chamber slide in 300 µL of DMEM with 10% FBS (antibiotic free) and incubated overnight at 37 °C with 5% CO_2. When cells reached 80-90% confluency, transfection reagents were prepared with 400 ng of plasmid, using 0.8 µL of P3000 and 0.8 µL of Lipofectamine 3000 (Thermo Fisher), diluted in Opti-MEM (Thermo Fisher) transfection media to a total volume of 20 µL. The transfection solution was scaled up according to the desired number of transfected wells. Cells were incubated at 37 °C with 5% CO_2 overnight. After overnight incubation, cells were washed three times with 200 µL of wash buffer (DPBS containing 5 mM $MgCl_2$ and 4.5 g/L glucose). Labeling was performed by incubating with 100 µL of the sgc8c(27)-**1** solution at the indicated concentration in binding buffer (DPBS containing 5 mM $MgCl_2$, 4.5 g/L glucose, and 0.1 mg/mL yeast tRNA) for 1 h, unless otherwise specified, at 37 °C with 5% CO_2. Cells were washed three more times with 200 µL of wash buffer and incubated with a 100 µL solution of 10 µg/mL neutravidin-tetramethylrhodamine (NA-TMR, Fisher, A6373A) in binding buffer for 5 minutes at room temperature. Cells were washed three more times with 200 µL of wash buffer. For label stability studying, cells were washed once with 100 µL of wash buffer and incubated in 250 µL of DMEM with 10% FBS for the indicated time. Cells were then washed three more times with 100 µL of wash buffer, and incubated with NA-TMR (10 µg/mL) in binding buffer for 5 minutes at room temperature. Live Cell Imaging Solution (200 µL, Invitrogen) was added and cells were imaged using a Zeiss Axio Observer Z1 with an Andor Zyla 4.2 camera, Plan-Apochromat 63x/1.4 objective, and the CFP (47 HE, ex. BP 436/20, em. BP 480/40) and TRITC (Chroma filter 49004, ex. ET 445/25, em. ET 605/70) filter sets. Internalization experiments were performed using an incubated stage (Tokai Hit) to maintain 37 °C and 5% CO_2. Images were exported in TIFF format using SlideBook. Image processing was conducted using ImageJ.

Flow cytometry analysis. Suspension cells (500,000 per condition) were pelleted via centrifugation (500 g, 5 min) and washed with 500 µL of wash buffer (DPBS containing 5 mM $MgCl_2$ and 4.5 g/L glucose). Adherent cell lines were incubated in 1 mL TrypLE Express Enzyme (Gibco) for 5 minutes at 37 °C with 5% CO_2 to lift them from a 10 cm plate, then diluted to 10 mL with the appropriate media, and counted. The 500,000 cells per condition were subjected to the same procedure as the suspension cells. Cells were then resuspended in 100 µL binding buffer (DPBS containing 5 mM $MgCl_2$, 4.5 g/L glucose, and 0.1 mg/mL yeast tRNA) containing indicated concentrations of sgc8c(27)-**1** in binding buffer and incubated for 1 h at 37 °C with 5% CO_2. Cells were then pelleted via centrifugation (500 g, 5 min) and washed with 200 µL wash buffer. To remove any non-covalently associated aptamer, cells were incubated in 100 µL TrypLE Express Enzyme (Gibco) for 10 minutes at 37 °C with 5% CO_2.[2] Wash buffer (900 µL) was added to the cells to quench the trypsin, and then cells were pelleted via centrifugation (500 g, 5 min). Cells were washed with wash buffer (200 µL) and resuspended in 100 µL of binding buffer containing 10 µg/mL SA-PE (streptavidin-phycoerythrin, Invitrogen) and incubated at room temperature for 5 minutes. Cells were again pelleted via centrifugation (500 g, 5 min) and washed 3x with 200 µL of wash buffer. Cells were resuspended in 200 µL of wash buffer and analyzed for red fluorescence using a CytoFLEX Flow Cytometer until 50,000 events were recorded. Results were analyzed using CytExpert software by normalizing mean fluorescence intensity to non-treated cells.

Supporting Figures & Table

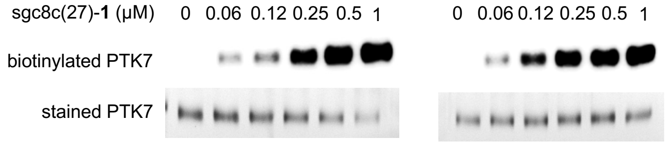

Figure S1. Western blot analysis of dose-dependent labeling of PTK7 by sgc8c(27)-**1**. Recombinant PTK7 (100 nM) was incubated with indicated concentrations of biotinylating aptamer for one hour at 37 °C in DPBS supplemented with 5 mM $MgCl_2$ and 4.5 g/L glucose (pH 7.4). Samples were analyzed by SDS-PAGE and visualized with SA-HRP. Total protein was visualized with a silver stain. For the quantification shown in **Figure 1B**, biotin band intensities were divided by the total protein, and then normalized to the highest intensity band (aptamer = 1 μM). Data points represent averages and error bars indicate standard deviation of two independent experiments.

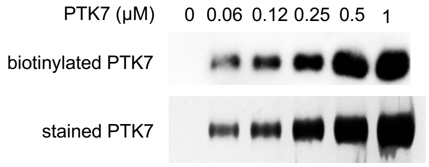

Figure S2. Titration of PTK7 for detection by sgc8c(27)-**1**. The biotinylating aptamer (1 μM) was incubated with increasing concentrations of PTK7 in DPBS supplemented with 5 mM $MgCl_2$ and 4.5 g/L glucose (pH 7.4) at 37 °C for one hour. Samples were analyzed by SDS-PAGE and visualized with SA-HRP. Total protein was visualized with a silver stain.

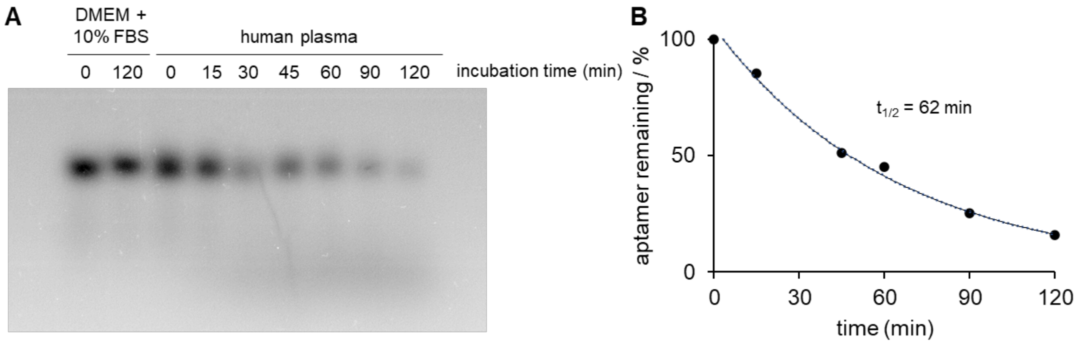

Figure S3. A) Degradation of ^{32}P-labeled sgc8c(27)-**1** in human plasma over time through Native-PAGE separation and radiography. B) Quantification of the percentage aptamer remaining, determined by band intensity (ImageJ).

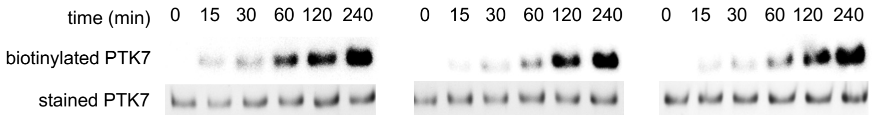

Figure S4. Western blot analysis of a labeling timecourse in triplicate. Recombinant PTK7 (100 nM) was incubated with sgc8c(27)-**1** (250 nM) for the indicated time at 37 °C in DPBS supplemented with 5 mM $MgCl_2$ and 4.5 g/L glucose (pH 7.4). Samples were analyzed by SDS-PAGE and visualized with SA-HRP. Total protein was visualized with a silver stain. For quantification (see **Figure 2E**), biotin band intensities were divided by the total protein, and were then normalized to the highest intensity band (t = 240 min). Data points represent averages and error bars indicate standard deviation of three independent experiments.

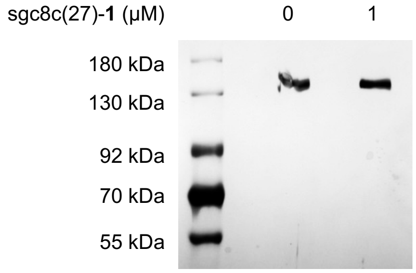

Figure S5. In-gel analysis of PTK7 and biotinylated PTK7 for MS analysis. PTK7 (100 nM) was incubated with 0 or 1 μM of aptamer for one hour at 37 °C in DPBS supplemented with 5 mM $MgCl_2$ and 4.5 g/L glucose (pH 7.4). Protein was visualized with silver stain and bands were excised and submitted to the Wayne State Proteomic Facility for MS analysis.

A

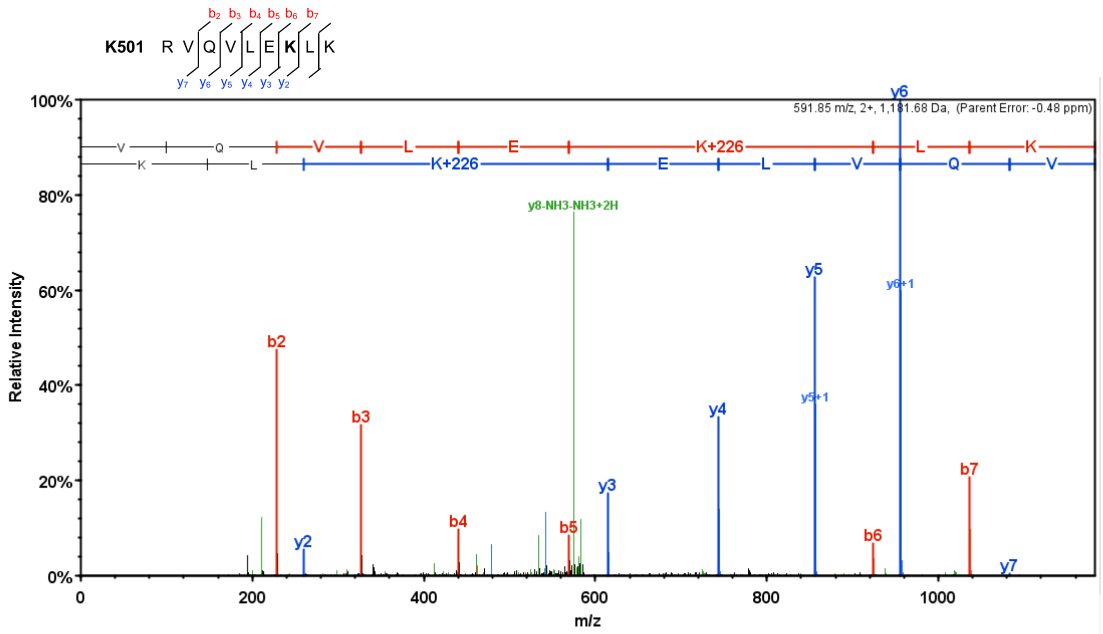

B

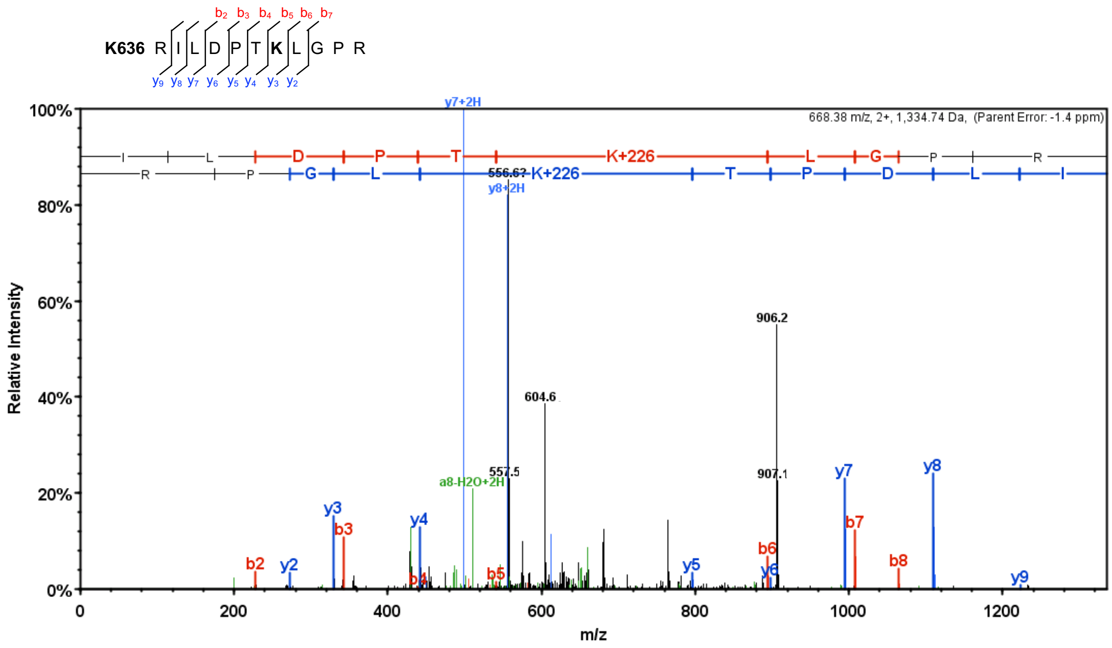

Figure S6. Peptide spectra from MS/MS experiments. A) K501 peptide spectra showing the unambiguous fragment ions for lysine biotinylation (K+226) in both the Y and B series ions. B) K636 peptide spectra showing the unambiguous fragment ions for lysine biotinylation in both the Y and B series ions.

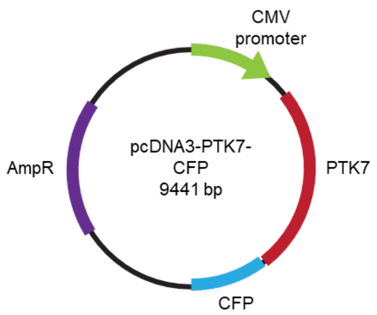

Figure S7. Plasmid map of pcDNA3-PTK7-CFP for mammalian cell expression.

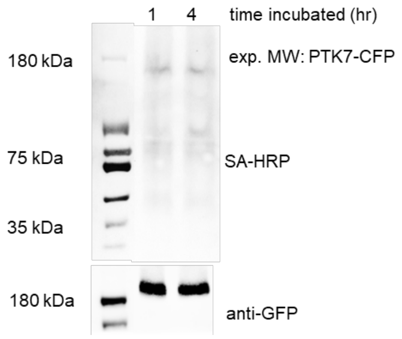

Figure S8. SA-HRP and anti-GFP western blots of lysate from HEK293T cells transfected with pcDNA3-PTK7-CFP and treated with the biotin-transferring thrombin aptamer TBA(3)-**1** (250 nM) for 1 h.[3]

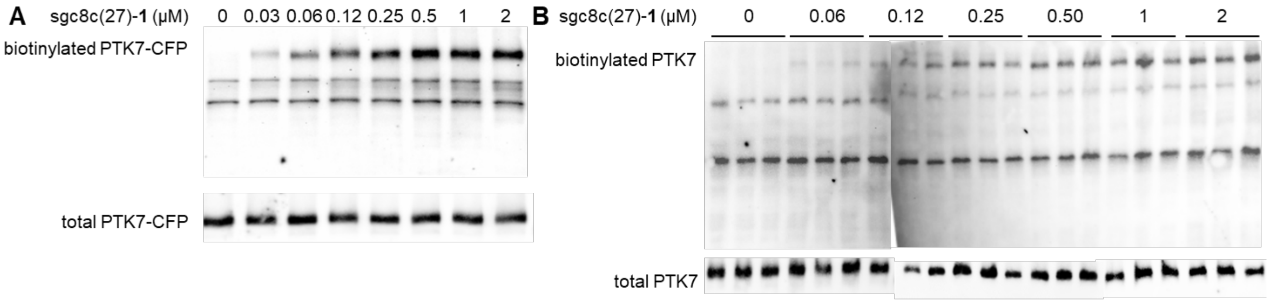

Figure S9. A) Full SA-HRP and anti-GFP western blot from **Figure 4B**. B) SA-HRP and anti-GFP western blot of lysate from HEK293T cells transfected with pcDNA3-PTK7-CFP and treated with increasing amounts of of sgc8c(27)-**1** for 1 h. For the quantification shown in **Figure 4B**, band intensities were determined by drawing a box around the entire lane and using the “plot lane” function in Image. Band intensities of the labeled proteins were divided by the band intensities of total protein, and were then normalized to the highest intensity band (aptamer = 2 μM). Data points represent averages and error bars indicate standard deviation of two independent experiments.

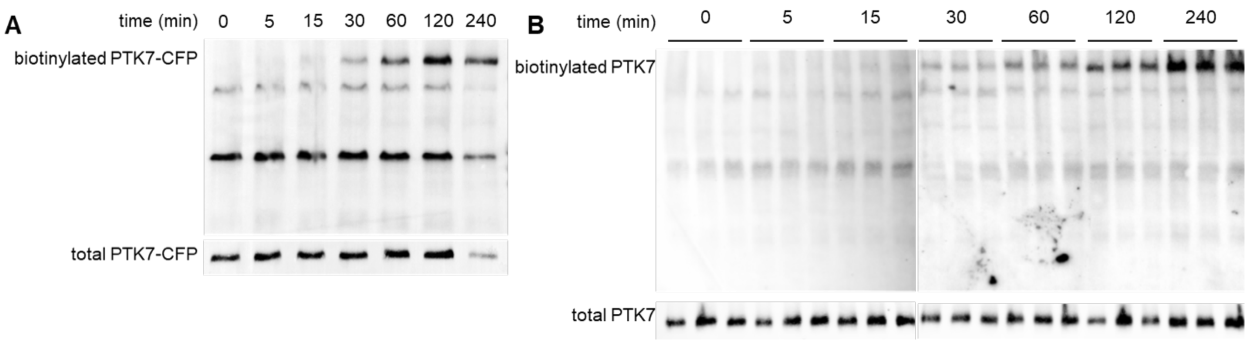

Figure S10. A) Full SA-HRP and anti-GFP western blots from **Figure 4C**. B) SA-HRP and anti-GFP western blots of lysate from HEK293T cells transfected with pcDNA3-PTK7-CFP and treated with 250 nM of sgc8c(27)-**1** for increasing amounts of time. For the quantification shown in **Figure 4C**, band intensities were determined by drawing a box around the entire lane and using the "plot lane" function in Image. Band intensities of the labeled proteins were divided by the band intensities of total protein, and were then normalized to the highest intensity band (incubation time = 240 min). Data points represent averages and error bars indicate standard deviation of two independent experiments.

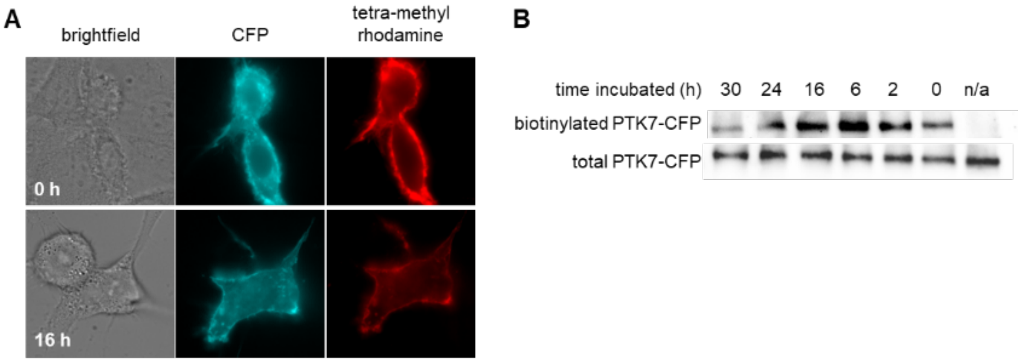

Figure S11. A) Images of NIH3T3 cells expressing PTK7-CFP incubated with sgc8c(27)-**1** for 1 h, followed by staining with NA-TMR either instantaneoulsy (0 h) or after 16 h. B) SA-HRP and anti-GFP western blot analysis of the persistence of biotinylated PTK7 over time. HEK293T cells transiently expressing PTK7-CFP were incubated with sgc8c(27)-**1** (250 nM) for 1 hour at 37 °C in DPBS supplemented with 5 mM $MgCl_2$ and 4.5 g/L glucose (pH 7.4). Cells were washed once with DPBS and incubated with DMEM containing 10% FBS and 1 µM unmodified sgc8c for the indicated time. Cell lysates were analyzed by SDS-PAGE.

Supporting Table S1. List of primers used to generate DNA constructs.

**Primer**	**Sequence (5' to 3')**
P1	AACCGGCTGGGCAAGAAGGATCCACCGGTCGCC
P2	CGCTAGCTCGAGTTACTTGTACAGCTCGTCCATGC
P3	GACGAGCTGTACAAGTAACTCGAGCTAGCGGCC
P4	GACCGGTGGATCCTTCTTGCCCAGCCGGTTCATC
P5	CGTCGCCGTCCAGCTCGACCA
P6	TAGAAGGCACAGTCGAGG

References

(1) Gibson, D. G.; Young, L.; Chuang, R. Y.; Venter, J. C.; Hutchison, C. A., 3rd; Smith, H. O. Enzymatic assembly of DNA molecules up to several hundred kilobases. *Nat Methods* **2009**, *6* (5), 343-345. DOI: 10.1038/nmeth.1318

(2) Xiao, Z.; Shangguan, D.; Cao, Z.; Fang, X.; Tan, W. Cell-specific internalization study of an aptamer from whole cell selection. *Chemistry* **2008**, *14* (6), 1769-1775. DOI: 10.1002/chem.200701330

(3) Tivon, Y.; Falcone, G.; Deiters, A. Protein Labeling and Crosslinking by Covalent Aptamers. *Angew Chem Int Ed Engl* **2021**, *60* (29), 15899-15904. DOI: 10.1002/anie.202101174.
